# Evaluation of the Hypoglycemic Activity of *Cucumis metuliferus* (Cucurbitaceae) Fruit Pulp Extract in Normoglycemic and Alloxan-Induced Hyperglycemic Rats

**DOI:** 10.4103/0975-1483.71633

**Published:** 2010

**Authors:** NS Jimam, NN Wannang, S Omale, B Gotom

**Affiliations:** *Department of Clinical Pharmacy, Faculty of Pharmaceutical Sciences, University of Jos, Jos, Nigeria*; 1 *Department of Pharmacology, Faculty of Pharmaceutical Sciences, University of Jos, Jos, Nigeria*

**Keywords:** Alloxan, *Cucumis metuliferus*, Hyperglycemic, Normoglycemic rats

## Abstract

The hypoglycemic effects of the fruit extract of *C. metuliferus* was investigated in normoglycemic and alloxan-induced hyperglycemic rats. The results showed that there was an insignificant (*P* > 0.05) decrease in the blood glucose concentration of normoglycemic rats treated with oral doses of 1000 and 1500 mg/kg of the extract. On the other hand, 500 mg/kg of the fruit extract produced an insignificant (*P* > 0.05) decrease in blood glucose levels of alloxan-treated rats, while 1000 and 1500 mg/kg oral dose points produced a significant (*P* < 0.05) decrease in the blood glucose concentration of hyperglycemic rats comparable to that produced by tolbutamide. From this study, the data suggested that the fruit extract did not alter the BGC level in normoglycemic rats, but had a potential hypoglycemic property in alloxan-induced hyperglycemic rats.

## INTRODUCTION

Diabetes Mellitus (DM) is an endocrine metabolic disorder in which the body does not produce sufficient insulin or lack of responsiveness to insulin, resulting in hyperglycemia (high blood glucose level). The classical symptoms include polyuria, polydipsia, weight loss, lethargy, polyphagia, visual blurring, frequent or recurring infections, cuts and bruises that are slow to heal, tingling and / or numbness in hands and / or feet, drowsiness, nausea, and decreased endurance during exercise.[1,2]

Despite the advancement in the understanding and management of DM, the incidence of the disease continues to increase.[3,4] More rural diabetic patients are now relying much more on traditional remedies,[5,6] due to the frequent claims by herbal medicine practitioners with regard to the effectiveness of herbal medicines in diabetes mellitus. Many of the commonly used phytomedicines have been evaluated by scientists for anti-diabetic activity.[7,8]

*Cucumis metuliferus* (Cucurbitaceae) is a monocious, climbing, annual herb that can be grown practically anywhere, provided the season is warm.[9] It flowers from July to September and the fruit ripens from October to December.[10] Many herbal practitioners on the plateau and environs use *C. metuliferus* fruit for the treatment of many ailments, including diabetes mellitus, hypertension, and typhoid fever. The fruit and leaves of this plant are widely consumed as a source of vegetables, with high nutritional values.[11] The anthelmintic properties of the plant seeds have also been documented.[12]

In the present study, we investigated the antidiabetic activity of *C. metuliferus* fruit extract in normal and alloxan-induced hyperglycemic rats.

## MATERIALS AND METHODS

### Plant materials

The ripe fruits of the non-bitter form of *C. metuliferus* were collected from Farin Gada and Babale area of Jos, Plateau State, Nigeria, in September, 2008. The whole plant and fruits were identified and authenticated by Prof. D. L. Wonang of the Department of Botany, University of Jos, Nigeria.

### Preparation of the extract

The ripe fruits collected from the plant were cut transversely using a blade, and the mesocarp contents were carefully scooped out from the pericarp with the aid of a spatula. The fleshy content was blended using an electric blender and the fluid product of the blending was passed through a sieve to separate the seeds from the juicy contents. The smooth filtrate was evenly spread on an aluminium tray and dried in a drying cabinet, at about 58°C until the liquid content had evaporated. The resultant product was air dried for several hours and then pounded to powder using a mortar and pestle. The powder was weighed and stored at room temperature in an air tight bottle, prior to use.

### Chemicals

Alloxan monohydrate (B. D. H. Laboratories, Chemical Division Poole, England) and Tolbutamide powder (Hoechst Fedco Pharma Ltd.) were used.

### Experimental animals

The Wister strains of male albino rats weighing between 150 – 240 g were obtained from the animal House of the University of Jos, Nigeria. They were fed with standard food (Pfizer Feed, Lagos) and water *ad libitum*.

### Activity of the extract in normoglycemic rats

Twenty-five (25) young, healthy, male albino rats were divided into five groups of five rats each. The baseline blood glucose concentration levels of the overnight fasted rats were measured before administration of the fruit extract. The control animals received equi-volume of distilled water (group 1), while the reference group (group 2) received 200 mg/kg of tolbutamide, through the oral route. Groups 3, 4, and 5 were orally administered 500, 1000, and 1500 mg/kg doses of *C. metuliferus* fruit extract, respectively.

After treatment, the blood glucose concentration (BGC) levels of the rats were measured at half, 1, 2, 4, 8, and 12 hours. The BGC levels were determined using the glucose-oxidase peroxidase method (Glucose assay kit) as expressed in mg/dl.

### Activity of the extract in alloxan-induced hyperglycemic rats

Twenty-five male Wister strains of the overnight fasted albino rats were made hyperglycemic by injecting alloxan monohydrate dissolved in distilled water at a dose of 120 mg/kg body weight, via the intraperitoneal route. After one hour of alloxan administration, the rats were given food and water *ad libitum*. The blood glucose concentration levels of the rats was measured after 72 hours of alloxan injection using a glucose assay kit, to determine the presence and level of hyperglycemia.

Animals in groups 1 and 2 were administered distilled water and 200 mg/kg tolbutamide, respectively. Animals in groups 3, 4, and 5 were treated with 500, 1000, and 1500 mg/kg dose of the extract, respectively. After treatment, the blood glucose concentration level was measured at half, 1, 2, 4, 8, and 12 hours using the glucose assay kit.

### Statistical analysis

Data obtained were subjected to one way ANOVA followed by the student’s t-test to determine the level of significance at *P* < 0.05 probability level.

## RESULTS

The oral administration of graded doses (500, 1000, and 1500 mg/kg) of *C. metuliferus* fruit extract produced insignificant (*P* > 0.05) transient decrease in BGC (blood glucose concentration), in normoglycemic rats, at dose points of 1000 and 1500 mg/kg, as shown in Table 1.

**Table 1:** Effect of oral administration of single graded doses of *C. metuliferus* fruit extract on blood glucose concentration in Normoglycemic rats

Dose (mg/kg) Time (hour)	Blood glucose concentration (mean ± SEM) mg/dl						
	o	½	1	2	4	8	12
Water	61.4 ± 4.2	69.0 ± 48	68.0 ± 3.6	57.4 ± 2.3	50.4 ± 2.7	53.0 ± 4.6	53.0 ± 4.6
Tolbutamide							
(200)	56.6 ± 2.2	39.4 ± 2.1	40.6 ± 30	39.0 ± 1.7	29.0 ± 3.6	21.8 ± 3.4	21.8 ± 3.4
500	52 ± 2.5	64.4 ± 3.8	60.8 ± 0.7	61.2 ± 3.0	45.2 ± 3.9	52.4 ± 5.0	52.4 ± 5.0
1000	64.6 ± 6.4	2.2 ± 5.9	71.4 ± 2.1	63.4 ± 3.4	51.0 ± 3.5	48.6 ± 5.0	46.8 ± 4.0
1500	64.0 ± 4.3	66.6 ± 6.8	60.2 ± 2.3	58.2 ± 1.1	53.4 ± 1.9	44.0 ± 1.4	47.2 ± 2.6

n = 5

From Table 2, it can be seen that graded doses (500, 1000, and 1500 mg/kg) of *C. metuliferus* fruit extract produced a significant (*P* < 0.05) decrease in BGC levels in alloxan-induced hyperglycemic rats when compared to the control hyperglycemic rats.

**Table 2:** Effect of oral aAdministration of single graded doses of *C. metuliferus* fruit extract on blood glucose concentration in Alloxan-induced hyperglycemic rats

Dose (mg/kg) Time (hour)	Blood glucose concentration (mean ± SEM) mg/dl						
	o	½	1	2	4	8	12
Water	130.0 ± 6.8	129.5 ± 7.3	129.2 ± 4.2	120.5 ± 3.2	132.3 ± 1.3	140.3 ± 3.7	138.0 ± 7.1
Tolbutamide							
(200)	280.9 ± 1.8	266.4 ± 1.5	249.0 ± 6.2	204.2 ± 2.5	129.8 ± 1.6	103.2 ± 2.8	91.4 ± 2.0
500	249.8 ± 2.6	194.6 ± 1.1	201.8 ± 9.3	129.5 ± 9.6	129.5 ± 0.8	122.3 ± 1.5	115.3 ± 2.1
1000	126.0 ± 3.2	119.3 ± 1.3	93.7 ± 7.9*	103.7 ± 1.7	94.7 ± 8.4	90.0 ± 2.7*	85.0 ± 9.1*
1500	131.2 ± 8.3	111.2 ± 4.2	105.2 ± 6.3*	110.4 ± 1.9	100.4 ± 1.6	98.6 ± 0.8*	91.4 ± 5.7*

n = 5

* *P* < 0.05

## DISCUSSION

The extract produced a significant decrease in glucose levels in the alloxan-induced rats and an insignificant decrease in the normal rats [Tables 1 and 2]. It is possible that the fruit extract improved the ability of the animals to utilize the external glucose load, a mechanism of action similar to that of the standard reference drug (tolbutamide), a sulfonylurea.[13] Sulfonylurea compounds produce hypoglycemia in normal animals by stimulating the pancreatic beta-cells to produce more insulin and by increasing the glycogen deposition in the liver, but are not effective in alloxan-induced diabetic animals because alloxan treatment causes a permanent destruction of the beta-cells.[14] In this study, a contrary result was obtained. The extract did not decrease the BGC levels in normoglycemic rats, but decreased it in hyperglycemic rats. This implies that in addition to the mechanism mentioned above, it is possible that other mechanisms are also involved. On the other hand, as exogenously administered insulin produces hypoglycemia in both normal and alloxan-induced diabetic subjects,[14,15] it would seem reasonable to suggest that the hypoglycemic principles in the *C. metuliferus* fruit extract exert a direct effect on diabetic rats, in a fashion similar to insulin. It has also been documented that saponin glucosides have a glucagon lowering effect,[16] which may enhance glucose utilization as indicated by the increase in hepatic glycogen. The presence of phytochemical materials, which may be responsible for the pharmacological activity has been shown by Jimam in 2008.[17]

## CONCLUSION

Oral treatment of experimental rats with *C. metuliferus* fruit extract caused significant (*P* < 0.05) reduction in the blood glucose level in alloxan-induced hyperglycemic rats, although there was no significant effect on the normoglycemic rats.

Though our results seem to justify the claim of the traditional healers, work is on-going to confirm the hypoglycemic activity of constituents and to evaluate its potential in the treatment of diabetes mellitus.
